# A phase I/II study of epertinib plus trastuzumab with or without chemotherapy in patients with HER2-positive metastatic breast cancer

**DOI:** 10.1186/s13058-019-1178-0

**Published:** 2019-12-31

**Authors:** Iain R. Macpherson, Pavlina Spiliopoulou, Saeed Rafii, Matilde Saggese, Richard D. Baird, Javier Garcia-Corbacho, Antoine Italiano, Jacques Bonneterre, Mario Campone, Nicola Cresti, John Posner, Yousuke Takeda, Akinori Arimura, James Spicer

**Affiliations:** 1 Institute of Cancer Sciences, University of Glasgow, Glasgow, UK; 2 Sarah Cannon Research Institute UK, London, UK; 3 Cancer Research UK Cambridge Centre, Cambridge, UK; 4 Institut Bergonie, Bordeaux, France; 5 Centre Oscar Lambret, Lille, France; 6 Institut de cancérologie de l’Ouest Site René Gauducheau, Saint Herblain, France; 7 Newcastle upon Tyne and Sir Bobby Robson Cancer Trials Research Centre, Freeman Hospital, Newcastle University, Newcastle Upon Tyne, UK; 8 Shionogi & Co. Ltd., Osaka, Japan; 9 School of Cancer and Pharmaceutical Sciences, King’s College London, Guy’s Hospital, 3rd Floor, Bermondsey Wing, St Thomas Street, London, SE1 9RT UK

**Keywords:** Epertinib, EGFR, HER2, HER2-positive breast cancer, Trastuzumab

## Abstract

**Background:**

Epertinib (S-222611) is a potent reversible inhibitor of HER2, EGFR and HER4. This trial evaluated the safety, tolerability, pharmacokinetics and antitumour activity of daily oral epertinib combined with trastuzumab (arm A), with trastuzumab plus vinorelbine (arm B) or with trastuzumab plus capecitabine (arm C), in patients with HER2-positive metastatic breast cancer (MBC).

**Methods:**

Eligible patients, with or without brain metastases, had received prior HER2-directed therapy. A dose-escalation phase determined the tolerability of each combination and established a dose for further study. Further, patients were recruited to expansion cohorts in each of the 3 arms to further explore efficacy and safety.

**Results:**

The recommended doses of epertinib were 600 mg, 200 mg and 400 mg in arms A, B and C, respectively. The most frequent grade 3/4 adverse event (AE) was diarrhoea in all arms, which was manageable with medical intervention and dose modification. The objective response rate (complete response [CR] plus partial response [PR]) in heavily pre-treated HER2-positive MBC patients at the recommended doses of epertinib combined with trastuzumab was 67% (*N* = 9), with trastuzumab plus vinorelbine was 0% (*N* = 5) and with trastuzumab plus capecitabine was 56% (*N* = 9). Notably, 4 of 6 patients previously treated with T-DM1 responded in the arm A expansion cohort (epertinib plus trastuzumab). In the arm C expansion cohort (epertinib plus trastuzumab plus capecitabine), 4 of 7 patients responded despite previous exposure to capecitabine. Measurable regression of brain metastases was observed in patients with CNS target lesions treated in both arms A and C.

**Conclusion:**

We observed safety, tolerability and encouraging antitumour activity of epertinib combined with trastuzumab, or with trastuzumab plus capecitabine. This supports further evaluation of these combinations in patients with pre-treated HER2-positive MBC, with or without brain metastases.

**Trial registration:**

EudraCT Number: 2013-003894-87; registered 09-September-2013.

**Electronic supplementary material:**

The online version of this article (10.1186/s13058-019-1178-0) contains supplementary material, which is available to authorized users.

## Background

Overexpression of the human epidermal growth factor receptor 2 (HER2) is found in 15–20% of patients with breast cancer and denotes an aggressive subtype of the disease [1–3]. Outcomes have improved substantially with the development of trastuzumab and subsequently other anti-HER2 agents including pertuzumab, ado-trastuzumab emtansine (T-DM1) and lapatinib. Metastatic HER2-positive cancer will often respond to sequential lines of HER2-targeted therapy, indicating an ongoing dependence on HER2 signalling. Hence, current management protocols comprise first-line trastuzumab and pertuzumab with taxane [4], and second-line T-DM1 [5]. Lapatinib and capecitabine are considered an option in the third-line setting [6]. Given the development of resistance to existing drugs, and the ongoing dependence on HER2, there is a potential role for additional HER2-targeting therapies. Furthermore, there remains an unmet need for drugs with greater penetration of the central nervous system (CNS).

Epertinib (S-222611, Shionogi & Co. Ltd., Osaka, Japan) is an orally active, reversible, selective and potent inhibitor of the epidermal growth factor receptor (EGFR), HER2 and HER4 receptor tyrosine kinases. Compared with lapatinib, epertinib showed more prolonged inhibition of phosphorylation of EGFR and HER2 in vitro, and 4–6-fold greater antitumour potency in mouse xenograft models [7]. Superior survival was also observed in a brain metastasis model of breast cancer, with good penetration to CNS tumours [7, 8]. A phase I study conducted in patients with various solid tumours, driven by EGFR and/or HER2, with or without brain metastases, demonstrated that once-daily dosing of epertinib monotherapy at 800 mg was well-tolerated [9, 10]. Promising antitumour activity was also observed, particularly in HER2-positive metastatic breast cancer (MBC), including partial regression of brain metastases [9, 10].

In this study, we evaluated the safety, tolerability, pharmacokinetics (PK) and efficacy of epertinib in combination with trastuzumab, or with trastuzumab plus chemotherapy (either vinorelbine or capecitabine). We recruited heavily pre-treated patients with HER2-positive breast cancer, with or without brain metastases, aiming to determine the recommended dose of epertinib in each combination and to select the most promising regimen for future clinical studies.

## Methods

### Patients

Eligible patients were ≥ 18 years old with histologically and/or cytologically confirmed HER2-positive metastatic breast cancer who had previously been treated with any anti-HER2 therapy and then progressed. To assess expression of HER2, fresh tumour biopsies or archival tissue were used, and HER2 status could be confirmed by either central or local assessment. Other eligibility criteria included measurable disease as per Response Evaluation Criteria In Solid Tumours (RECIST) v1.1, an Eastern Cooperative Oncology Group (ECOG) performance status of 0 or 1, and adequate bone marrow, renal, hepatic and left ventricular function. Patients who had previously received vinorelbine and/or capecitabine were eligible, as were patients with brain metastases. Concomitant medication with strong CYP3A4 inhibitors/inducers, or with CYP3A4 substrates with a narrow therapeutic index, was prohibited. This study was approved after review by the relevant regulatory and independent ethics committees (EudraCT Number: 2013-003894-87) and was conducted in accordance with the Declaration of Helsinki and International Conference on Harmonization Good Clinical Practice. All patients provided written informed consent before enrolment.

### Study design and drug administration

This phase I/II, multi-centre, open-label study comprised dose-escalation and expansion components to evaluate safety, tolerability, PK and preliminary antitumour activity of epertinib administered orally once a day in combination with trastuzumab (arm A), trastuzumab plus vinorelbine (arm B) or trastuzumab plus capecitabine (arm C). Epertinib oral dosing started on day 3 of cycle 1 (after 48 h PK blood sampling for capecitabine or vinorelbine) and then administered continuously. The protocol-specified duration of study treatment was 36 weeks unless there was radiographically documented disease progression or dose-limiting or intolerable toxicity. After 36 weeks participants who were benefiting could continue to receive epertinib via a separate named patient program.

In the dose-escalation component, a modified ‘3 + 3’ design was used to assess the safety and tolerability of each combination. The dose of epertinib was escalated from the initial dose (400 mg in arm A and 200 mg in arms B and C) provided at least 3 subjects had completed 21 days’ treatment with no dose-limiting toxicity (DLT). If any one of the first 3 subjects experienced DLT, up to 4 further subjects were enrolled at that dose level, with dose escalation proceeding if at least 6 subjects completed 21 days’ treatment with no more than 1 DLT. Protocol-specified DLTs included uncomplicated grade 4 neutropenia for ≥ 7 days or neutropenia of any duration associated with fever > 38.5 °C, grade 3 thrombocytopenia associated with bleeding requiring platelet transfusion; grade 3 or 4 non-haematologic toxicity (except incompletely treated nausea, vomiting, or diarrhoea); persistent grade ≥ 2 diarrhoea or nausea and/or grade > 1 vomiting for 7 or more days despite supportive care; and a decline in LVEF by ≥ 10% from baseline.

Trastuzumab was given intravenously at 8 mg/kg as an initial dose and subsequently 6 mg/kg, or at 600 mg/5 mL fixed dose subcutaneously, once every 3 weeks. Patients in arm B additionally received vinorelbine 60 mg/m^2^ orally on days 1 and 8 of a 21-day cycle, and patients in arm C received capecitabine 1000 mg/m^2^ orally twice daily for 14 days followed by a 7-day rest period (21-day cycles). Loperamide could be used to treat diarrhoea but was not used for prophylaxis. Arms were expanded up to a further 9 evaluable patients to obtain confirmatory tolerability and PK data to determine a recommended dose for future clinical studies.

All adverse events (AEs) were monitored and coded using National Cancer Institute (NCI) Common Terminology Criteria for Adverse Events (CTCAE) Version 4.0. Participants were treated until disease progression, treatment-emergent toxicities or withdrawal of consent.

### Pharmacokinetic analysis

Blood samples were assayed for PK profiles of the concomitant anticancer drugs (CADs) on 2 occasions, before (Day 1-3) and after (Day 22-24) introduction of epertinib. Dosing with epertinib commenced orally once per day on day 3, after the last blood sample of the first CDA PK profile. PK profiles of the CAD in presence of epertinib and epertinib (its active de-alkylated and lactam metabolites) were obtained when the patient had received at least 10 consecutive days of combination therapy. PK sampling was performed at the following times during treatment; pre dose, 1, 2, 4, 6, 8, 12, 24 and 48 hours post dose for analysis of vinorelbine, and pre dose, 0.5, 1, 2, 4, 6, 8, and 12 hours post dose for capecitabine and its major metabolites (5-fluorouracil and alpha-fluoro-beta-alanin). The blood samples on day 22 and 23 were analysed for PK analysis of epertinib, and its active de-alkylated and lactam metabolites (pre dose, 1, 2, 4, 6, 8, 12 and 24 hours post dose). 

### Tumour evaluation

Tumour response was assessed by each investigator at 6- to 9-week intervals according to RECIST v1.1. Patients who had at least one additional scan after the baseline or patients who had no additional scan but discontinued due to clinical disease progression were considered evaluable for response. The objective response rate (ORR), comprising the proportion of patients achieving complete response (CR) or partial response (PR), and clinical benefit rate (CBR; the proportion of patients achieving CR, PR or stable disease [SD] ≥ 6 months) were summarised.

## Results

A total of 45 patients with HER2-positive metastatic breast cancer were enrolled between August 2014 and November 2015 at 8 sites in the UK and France. Patient demographics and baseline characteristics are summarised in Table 1. Of the 45 patients enrolled, 38 (84%) had received at least 4 lines of prior anti-cancer therapy. All 45 patients (100%) had previously received trastuzumab, 31 (69%) had also received T-DM1 and 19 (42%) had received lapatinib. Patient disposition is summarised in Additional file 1: Figure S1.

**Table 1:** Patient demographics and baseline characteristics

	Arm A (*N*=21)	Arm B (*N*=7)	Arm C (*N*=17)	Overall (*N*=45)
	Epertinib + T	Epertinib + T + V	Epertinib + T + C	
Sex				
Female	21 (100%)	6 (85.7%)	17 (100%)	44 (97.8%)
Male	0	1 (14.3%)	0	1 (2.2%)
Age (years)				
Mean (range)	57.6 (38-79)	49.0 (36-57)	56.8 (36-75)	56.0 (36-79)
ECOG PS at screening				
0	11 (52.4%)	6 (85.7%)	11 (64.7%)	28 (62.2%)
1	10 (47.6%)	1 (14.3%)	6 (35.3%)	17 (37.8%)
Number of metastatic sites at screening				
1-3	7 (33.3%)	4 (57.1%)	5 (29.4%)	16 (35.6%)
≥4	14 (66.7%)	3 (42.9%)	12 (70.6%)	29 (64.4%)
Number of prior anti-cancer therapy regimens				
1-3	0	1 (14.3%)	6 (35.3%)	7 (15.6%)
≥4	21 (100%)	6 (85.7%)	11 (64.7%)	38 (84.4%)
Prior HER2-targeted therapy				
Trastuzumab	21 (100%)	7 (100%)	17 (100%)	45 (100%)
T-DM1	15 (71.4%)	3 (42.9%)	13 (76.5%)	31 (68.9%)
Lapatinib	10 (47.6%)	2 (28.6%)	7 (41.2%)	19 (42.2%)
Pertuzumab	3 (14.3%)	2 (28.6%)	4 (23.5%)	9 (20.0%)
Prior Capecitabine / Vinorelbine				
Capecitabine	16 (76.2%)	4 (57.1%)	11 (64.7%)	31 (68.9%)
Vinorelbine	9 (42.9%)	1 (14.3%)	10 (58.8%)	20 (44.4%)

*Abbreviations*: *T* trastuzumab, *V* vinorelbine, *C* capecitabine, *ECOG* Eastern cooperative oncology group, *PS* performance status

### Safety and tolerability

Adverse events occurring in at least 10% of patients are summarised in Table 2. Overall, the most frequently reported AEs were diarrhoea, nausea, increased bilirubin, decreased appetite and vomiting. Diarrhoea was generally managed with drugs such as loperamide and sometimes by holding and/or reducing the dose of epertinib. Grade 3 diarrhoea was reported in 16 patients (36%) with a median duration of 3 days and maximum duration of 8 days per event. Nausea and vomiting were generally low grade. Blood bilirubin elevation, previously reported with epertinib [9, 10], was not associated with elevation of liver enzymes or haematological abnormalities, except for one patient who experienced liver dysfunction which was subsequently shown to be due to progression of hepatic metastases. Neutropenia was observed in arm B (57%). The incidence of palmar-plantar erythrodysaesthesia was greatest in arm C (65%).

**Table 2:** Treatment-emergent adverse events occurring in ≥ 10% of patients

Grade	Arm A (*N*=21)		Arm B (*N*=7)		Arm C (*N*=17)		Overall (*N*=45)	
	Epertinib + T		Epertinib + T + V		Epertinib + T + C			
	all, n (%)	≥3, n (%)	all, n (%)	≥3, n (%)	all, n (%)	≥3, n (%)	all, n (%)	≥3, n (%)
Gastrointestinal disorders								
Diarrhoea	20 (95.2)	8 (38.1)	7 (100)	3(42.9)	16 (94.1)	5 (29.4)	43 (95.6)	16 (35.6)
Nausea	17 (81.0)	1 (4.8)	7 (100)	0	12 (70.6)	0	36 (80.0)	1 (2.2)
Vomiting	7 (33.3)	1 (4.8)	4 (57.1)	1 (14.3)	6 (35.3)	0	17 (37.8)	2 (4.4)
Stomatitis	6 (28.6)	0	0	0	5 (29.4)	0	11 (24.4)	0
Constipation	3 (14.3)	0	2 (28.6)	0	3 (17.6)	0	8 (17.8)	0
Abdominal Pain	4 (19.0)	0	1 (14.3)	0	2 (11.8)	0	7 (15.6)	0
Dyspepsia	1 (4.8)	0	1 (14.3)	0	3 (17.6)	0	5 (11.1)	0
Metabolism and nutrition disorders								
Decreased appetite	9 (42.9)	0	2 (28.6)	0	6 (35.3)	1 (5.9)	17 (37.8)	1 (2.2)
Hypokalaemia	4 (19.0)	0	1 (14.3)	1 (14.3)	6 (35.3)	3 (17.6)	11 (24.4)	4 (8.9)
General disorders								
Fatigue	5 (23.8)	0	3 (42.9)	1 (14.3)	6 (35.3)	0	14 (31.1)	1 (2.2)
Oedema peripheral	2 (9.5)	0	0	0	4 (23.5)	0	6 (13.3)	0
Pyrexia	2 (9.5)	0	2 (28.6)	0	2 (11.8)	0	6 (13.3)	0
Asthenia	1 (4.8)	0	0	0	4 (23.5)	0	5 (11.1)	0
Skin and subcutaneous tissue disorders								
Palmar-plantar erythrodysaesthesia syndrome	2 (9.5)	0	0	0	11 (64.7)	1 (5.9)	13 (28.9)	1 (2.2)
Rash	5 (23.8)	0	2 (28.6)	0	4 (23.5)	0	11 (24.4)	0
Pruritus	6 (28.6)	0	0	0	2 (11.8)	0	8 (17.8)	0
Dermatitis acneiform	6 (28.6)	0	0	0	0	0	6 (13.3)	0
Blood system disorders								
Anaemia	3 (14.3)	1 (4.8)	0	0	6(35.3)	0	9 (20.0)	1 (2.2)
Neutropenia	1 (4.8)	0	4 (57.1)	4 (57.1)	2(11.8)	1 (5.9)	7 (15.6)	5 (11.1)
Infections and infestations								
Paronychia	1 (4.8)	0	2 (28.6)	0	6 (35.3)	0	9 (20.0)	0
Upper respiratory tract infection	3 (14.3)	0	2 (28.6)	0	1 (5.9)	0	6 (13.3)	0
Urinary tract infection	2 (9.5)	0	0	0	4 (23.5)	0	6 (13.3)	0
Respiratory disorders								
Dyspnoea	2 (9.5)	1 (4.8)	1 (14.3)	0	5 (29.4)	1 (5.9)	8 (17.8)	2 (4.4)
Epistaxis	3 (14.3)	0	2 (28.6)	0	3 (17.6)	0	8 (17.8)	0
Cough	3 (14.3)	0	1 (14.3)	0	2 (11.8)	0	6 (13.3)	0
Nervous system disorders								
Headache	4 (19.0)	0	1 (14.3)	0	2(11.8)	0	7 (15.6)	0
Lethargy	2 (9.5)	0	3 (42.9)	0	1 (5.9)	0	6 (13.3)	0
Musculoskeletal disorder								
Muscle spasms	2 (9.5)	0	1 (14.3)	0	2 (11.8)	0	5 (11.1)	0
Eye disorder								
Dry eye	1 (4.8)	0	1 (14.3)	0	3 (17.6)	0	5 (11.1)	0
Investigations								
Blood bilirubin increased	10 (47.6)	1 (4.8)	2 (28.6)	0	10 (58.8)	3 (17.6)	22 (48.9)	4 (8.9)
ALT increased	3 (14.3)	0	5 (71.4)	0	2 (11.8)	1 (5.9)	10 (22.2)	1 (2.2)
AST increased	2 (9.5)	0	3 (42.9)	0	4 (23.5)	1 (5.9)	9 (20.0)	1 (2.2)
Weight decreased	2 (9.5)	0	0	0	3 (17.6)	0	5 (11.1)	0

*Abbreviations*: *T* trastuzumab, *V* vinorelbine, *C* capecitabine

In arm A, grade 3 diarrhoea during the first 21-day period of daily dosing with epertinib was observed in 4 of 7 patients at the 800-mg dose level. Although only 1 formal DLT was observed, this dose was not considered to be well-tolerated, and therefore, 600 mg was the dose recommended for further study of this combination with trastuzumab. Four of 9 patients in the 600 mg cohort had a dose reduction because of AEs, but no patients permanently discontinued treatment because of toxicity. In arm B, 200 mg was determined as the maximum tolerable dose (MTD) because 2 patients in the 400-mg cohort experienced DLTs (grade 4 neutropenia > 7 days, *N* = 2). No patient in the 200-mg cohort discontinued treatment because of AEs. In arm C, 600 mg was not considered to be well-tolerated because withdrawal of the study drug due to grade 3 diarrhoea lasting less than 7 days (*N* = 1), or blood bilirubin elevation (*N* = 1), was required in 2 of the 4 patients during cycle 1. Although these were not pre-defined DLTs, 400 mg was defined as the recommended dose. Two of 9 patients in the 400-mg cohort required dose reduction of epertinib because of AEs, but no permanent discontinuation was required. No grade 5 toxicity was observed across all arms.

### Pharmacokinetic analysis

No relevant differences between treatment arms, or in the presence and absence of concomitant drugs, were found in PK parameters for epertinib and its metabolites (Additional file 3: Table S1). Similarly, the presence of epertinib appeared to have no significant effect on the PK of vinorelbine and capecitabine (Additional file 3: Tables S2-S4). PK analysis was not performed in the 400-mg cohort in arm B due to temporary drug discontinuation for safety purposes during cycle 1.

### Antitumour activity

**Fig. 1:**
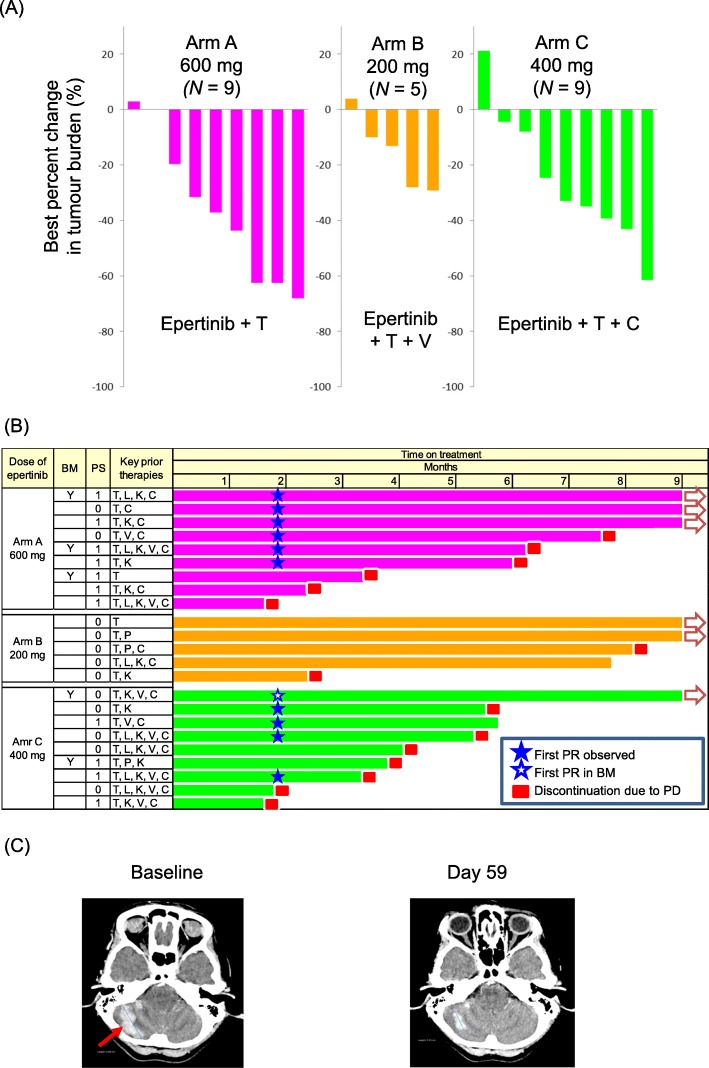
Antitumour activity (**a**) and duration on treatment (**b**) for patients in the recommended dose cohorts for each arm. Partial regression of brain metastases in a 53-year-old patient after 59 days of treatment with epertinib 400 mg in combination with trastuzumab plus capecitabine in arm C (**c**). Brain metastases first appeared during prior treatment with trastuzumab with capecitabine, and the patient underwent whole-brain radiotherapy followed by T-DM1. After further progression in the brain the patient was enrolled in this study. T: trastuzumab, L: lapatinib, K: T-DM1, P: pertuzumab, V: vinorelbine, C: capecitabine, BM: brain metastases

Forty-four of the 45 patients enrolled were evaluable for tumour response. One patient discontinued without any efficacy evaluation due to DLT at 800 mg in arm A. The tumour response for each arm and dose are summarised in Table 3. The magnitude of response and duration of treatment for patients receiving epertinib at the clinically recommended dose or MTD in arms A, B and C are summarised in Fig. 1 a and b.

**Table 3:** Antitumour activity in evaluable patients

Dose of epertinib	Arm A			Arm B		Arm C			
	Epertinib + T			Epertinib + T + V		Epertinib + T + C			
	400mg	600mg	800mg	200mg	400mg	200mg	400mg	600mg	Overall
	*N*=5	*N*=9	*N*=7	*N*=5	*N*=2	*N*=4	*N*=9	*N*=4	*N*=45
BOR									
CR	0	0	0	0	0	0	0	0	0
PR	0	6 (66.7%)	1 (14.3%)	0	2 (100%)	0	5 (55.6%)	2 (50.0%)	16 (35.6%)
SD ≥ 6 months	2 (40.0%)	0	1 (14.3%)	4 (80.0%)	0	1 (25.0%)	0	1 (25.0%)	9 (20.0%)
CBR	2 (40.0%)	6 (66.7%)	2 (28.6%)	4 (80.0%)	2 (100%)	1 (25.0%)	5 (55.6%)	3 (75.0%)	25 (55.6%)
PD	2 (40.0%)	1 (11.1%)	2 (28.6%)	0	0	1 (25.0%)	1 (11.1%)	1 (25.0%)	8 (17.8%)

Clinical benefit is defined as objective response plus SD at 6 months

*Abbreviations*: *T* trastuzumab, *V* vinorelbine, *C* capecitabine, *BOR* best overall response, *CR* complete response, *PR* partial response, *SD* stable disease, *CBR* clinical benefit rate, *PD* progressive disease

In arm A, the ORR at 600 mg epertinib was 67% (6/9 patients). Notably, 4 of 6 patients pre-treated with T-DM1, and 5 of 7 patients pre-treated with capecitabine, experienced PR in this cohort. Two of 3 patients with brain metastases achieved PR as assessed by RECIST v1.1 in this cohort. In arm B, since there was no tumour response in the first 5 patients treated with 200 mg and, given concerns that there may be insufficient exposure to epertinib at this dose, no additional patients were enrolled. However, the clinical benefit rate was 80%. In arm C, the ORR at 400 mg was 56% (5/9 patients). Interestingly, 4 of 7 patients previously treated with capecitabine experienced PR in this cohort. One of two patients with brain metastases showed partial regression of CNS lesions as assessed by RECIST v1.1 (Fig. 1c). Across cohorts, a reduction in the longest diameter was observed in 4 of 5 patients with CNS target lesions (Additional file 2: Figure S2). This included all patients treated in the recommended dose cohorts (arm A; *N* = 2 and arm C; *N* = 2).

## Discussion

In combination with trastuzumab, 600 mg daily of epertinib was defined as the recommended dose based on the safety data. The most frequently occurring AE in this cohort was diarrhoea, which could be managed by anti-diarrhoeal medication and/or dose reduction. At this dose, epertinib plus trastuzumab showed a remarkable response rate (67%) in patients heavily treated with regimens including trastuzumab, T-DM1, lapatinib, pertuzumab and chemotherapy. This may indicate greater efficacy for dual therapy with trastuzumab, given the 19% response rate that was observed with epertinib monotherapy at a higher dose of 800 mg in HER2-positive MBC [10]. Additional benefit for combination with trastuzumab over single agent epertinib, despite previous disease progression on a trastuzumab-containing regimen, would be consistent with observations with other HER2-directed tyrosine kinase inhibitors. For example, the combination of lapatinib plus trastuzumab was superior to lapatinib monotherapy in HER2-positive MBC that had progressed on trastuzumab in terms of median progression-free survival (PFS), although not response rate [11].

In clinical practice, trastuzumab is often continued beyond progression with a switch to an alternative combination chemotherapy agent, as this improves outcomes compared with chemotherapy alone [12]. Standard first-line therapy in MBC is trastuzumab plus taxane with or without pertuzumab, and the chemotherapy regimens tested with epertinib and trastuzumab in this trial where chosen with this standard of care in mind. In combination with trastuzumab plus vinorelbine, 200 mg of epertinib was defined as MTD because of grade 4 neutropenia [13]. There was no pharmacological interaction between epertinib 200 mg and vinorelbine based on plasma exposure, although a PK analysis could not be completed in the two patients treated in the 400 mg cohort because of drug cessation related to the DLTs. Since neutropenia has not been reported in patients receiving monotherapy with epertinib [9, 10], or observed in arms A or C our study, it is likely that it was attributable to the vinorelbine in arm B. At an epertinib dose of 200 mg in combination with trastuzumab plus vinorelbine, there were no tumour responses in the first 5 patients and so recruitment to this arm was halted.

Trastuzumab plus capecitabine combination therapy is considered to be effective in heavily pre-treated HER2-positive MBC [12, 14]. The PHEREXA study has demonstrated that the addition of pertuzumab to trastuzumab plus capecitabine did not significantly improve PFS [15]; objective response rates were 33% with trastuzumab plus capecitabine, and 41% with pertuzumab and trastuzumab plus capecitabine. Although we cannot draw direct comparisons, in our study the objective response rate with epertinib 400 mg in combination with trastuzumab plus capecitabine was 56%, despite prior capecitabine treatment in 7 of 9 patients, supporting further investigation of this regimen.

Capecitabine plus lapatinib has been approved in HER2-positive MBC previously treated with trastuzumab and is often favoured for patients with brain metastases [16]. Combining chemotherapy with HER2-directed therapy for the management CNS disease is further supported by the activity of capecitabine plus neratinib in patients with treatment-refractory brain metastases treated within the TBCRC 022 study [17]. The CNS response rate of 49% compared favourably to the 8% response rate that was previously reported for a TBCRC 022 neratinib monotherapy cohort [18]. In our study, prolonged disease stabilisation (≥ 6 months) was seen in 2 of 3 patients with brain metastases in arm A, and a partial response in brain metastases occurred in one of 2 patients in arm C. All patients had received prior radiotherapy and experienced subsequent CNS progression. We have recently reported that both epertinib and lapatinib accumulate to a comparable extent in tumour deposits of a rapidly growing mouse brain metastasis model. By contrast, only epertinib accumulates in brain metastases derived from a slow-growing tumour cell line, in which the blood-tumour barrier can be considered to be intact [8]. These pre-clinical observations, combined with our clinical findings, suggest that epertinib may be particularly effective in preventing or controlling brain metastases in patients.

## Conclusions

Combination therapy of epertinib with trastuzumab showed robust antitumour activity with manageable diarrhoea even in heavily pre-treated patients. Epertinib in addition to trastuzumab, with or without capecitabine, could be an effective regimen to treat refractory HER2-positive breast cancer, including those patients with CNS metastases, and merits further clinical evaluation.

## Additional files

Additional file 1: **Figure S1.** Patient disposition. (TIFF 7122 kb)

Additional file 2: **Figure S2.** Waterfall plot of maximum relative brain tumour reduction (as per RECISTv1.1) in comparison to baseline in all patients with CNS target lesions (*N* = 5). One column represents one patient. (TIFF 7122 kb)

Additional file 3: **Table S1.** Summary of Geometric Mean (Geometric CV%) Pharmacokinetics Parameters for Epertinib and its Metabolites with concomitant anticancer drugs (CADs). Table S2. Summary of Geometric Mean (Geometric CV%) Pharmacokinetics Parameters for Vinorelbine with and without Epertinib (Arm B). Table S3. Summary of Geometric Mean (Geometric CV%) Pharmacokinetics Parameters for Capecitabine with and without Epertinib (Arm C). Table S4. Summary of Geometric Mean (Geometric CV%) Pharmacokinetics Parameters for 5-Fluorouracil following treatment with Capecitabine with and without Epertinib (Arm C) (DOCX 52 kb)

## Data Availability

The datasets generated during and/or analysed during the current study are not publicly available in accordance with the Commission Guideline—Guidance on posting and publication of result-related information on clinical trials in relation to the implementation of Article 57(2) of Regulation (EC) No 726/2004 and Article 41(2) of Regulation (EC) No 1901/2006 (2012/C 302/03) Section 5, which states that result-related information on non-paediatric phase I clinical trials is not made public.

## Abbreviations

AE: Adverse event
ALT: Alanine aminotransferase
AST: Aspartate aminotransferase
BOR: Best overall response
CAD: Concomitant anti-cancer drug
CBR: Clinical benefit rate
CNS: Central nervous system
CR: Complete response
CTCAE: Common Terminology Criteria for Adverse Events
DLT: Dose-limiting toxicity
ECOG PS: Eastern Cooperative Oncology Group performance status
EGFR: Epidermal growth factor receptor
HER2: Human epidermal growth factor receptor 2
LVEF: Left ventricular ejection fraction
MBC: Metastatic breast cancer
MTD: Maximum tolerable dose
NCI: National Cancer Institute
ORR: Objective response rate
PD: Progressive disease
PFS: Progression-free survival
PK: Pharmacokinetics
PR: Partial response
RECIST: Response Evaluation Criteria In Solid Tumours
SAE: Serious adverse event
SD: Stable disease
