# Supplementary figures and images for: A phase I/II study of epertinib plus trastuzumab with or without chemotherapy in patients with HER2-positive metastatic breast cancer
Source: Breast Cancer Res. 2019 Dec 31;22:1. doi: 10.1186/s13058-019-1178-0 (PMC6938617; doi:10.1186/s13058-019-1178-0)

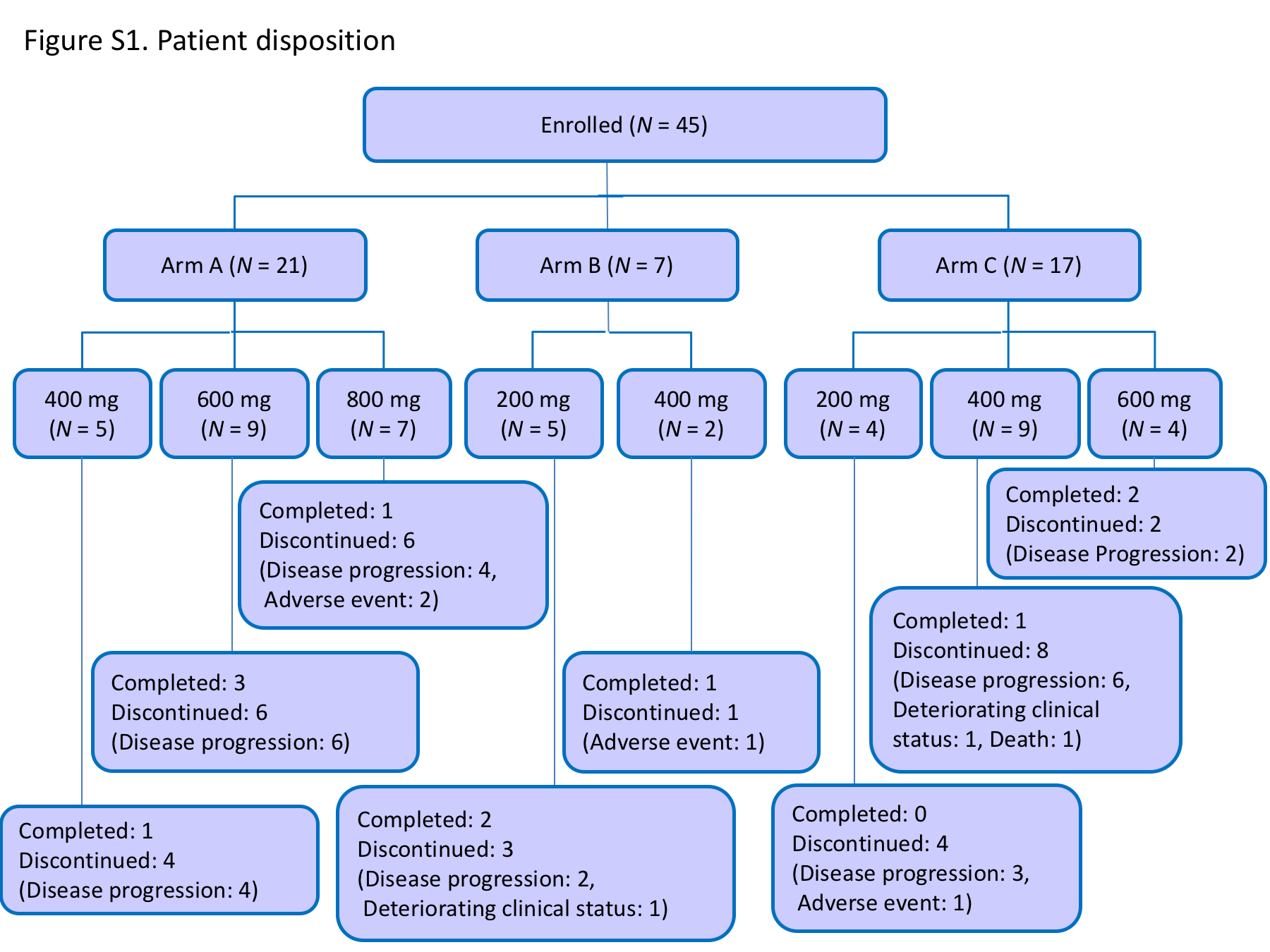

Supplement: Supplementary file 1 — Figure S1. Patient disposition. (TIFF 7122 kb) [file 13058_2019_1178_MOESM1_ESM.tiff]

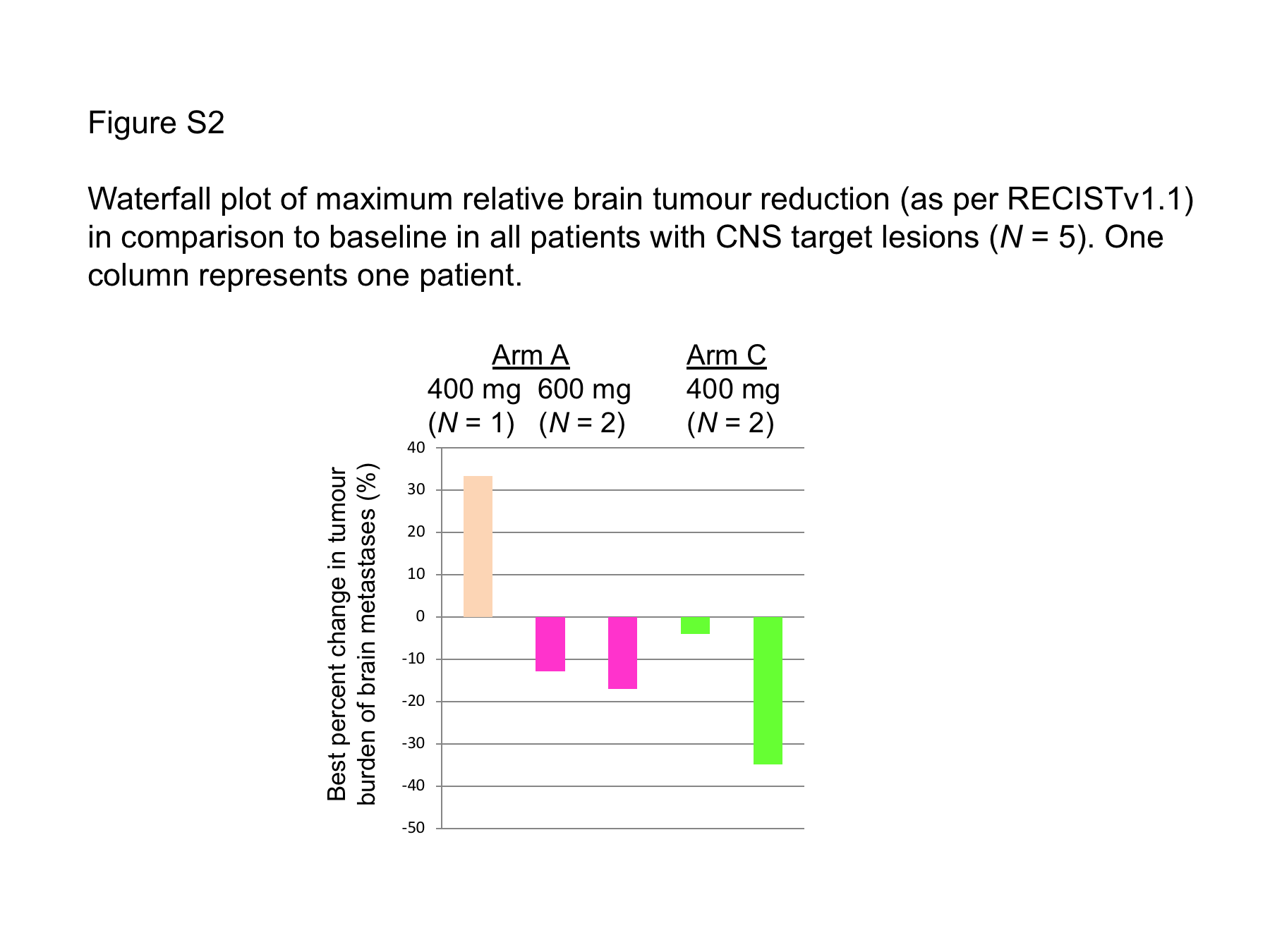

Supplement: Supplementary file 2 — Figure S2. Waterfall plot of maximum relative brain tumour reduction (as per RECISTv1.1) in comparison to baseline in all patients with CNS target lesions (N = 5). One column represents one patient. (TIFF 7122 kb) [file 13058_2019_1178_MOESM2_ESM.tiff]
