# Supplementary material for: A phase I/II study of epertinib plus trastuzumab with or without chemotherapy in patients with HER2-positive metastatic breast cancer
Source: Breast Cancer Res. 2019 Dec 31;22:1. doi: 10.1186/s13058-019-1178-0 (PMC6938617; doi:10.1186/s13058-019-1178-0)
Supplement: Supplementary file 3 — Table S1. Summary of Geometric Mean (Geometric CV%) Pharmacokinetics Parameters for Epertinib and its Metabolites with concomitant anticancer drugs (CADs). Table S2. Summary of Geometric Mean (Geometric CV%) Pharmacokinetics Parameters for Vinorelbine with and without Epertinib (Arm B). Table S3. Summary of Geometric Mean (Geometric CV%) Pharmacokinetics Parameters for Capecitabine with and without Epertinib (Arm C). Table S4. Summary of Geometric Mean (Geometric CV%) Pharmacokinetics Parameters for 5-Fluorouracil following treatment with Capecitabine with and without Epertinib (Arm C) (DOCX 52 kb) [file 13058_2019_1178_MOESM3_ESM.docx]

Table S1

Summary of Geometric Mean (Geometric CV%) Pharmacokinetics Parameters for Epertinib and its Metabolites with CADs

|  | **Arm A** | | | | | | **Arm B** | | | | **Arm C** | | | | | |
| --- | --- | --- | --- | --- | --- | --- | --- | --- | --- | --- | --- | --- | --- | --- | --- | --- |
| **Dose of epertinib** | ***N*** | **400 mg**  ***N* = 5** | ***N*** | **600 mg**  ***N* = 9** | ***N*** | **800 mg**  ***N* = 6** | ***N*** | **200 mg**  ***N* = 5** | ***N*** | **400 mg**  ***N* = 2** | ***N*** | **200 mg**  ***N* = 4** | ***N*** | **400 mg**  ***N* = 9** | ***N*** | **600 mg**  ***N* = 4** |
| **Epertinib** |  |  |  |  |  |  |  |  |  |  |  |  |  |  |  |  |
| T_ss, max_ ^a^ (hr) | 3 | 3.58  (2.75-8.00) | 5 | 6.12  (6.00-11.05) | 3 | 3.98  (3.97-4.02) | 5 | 3.93  (2.00-6.00) | 0 | NC | 3 | 5.97  (3.97-6.00) | 8 | 5.01  (2.00-11.58) | 2 | 3.93  (3.88-3.98) |
| C_ss, max_ (ng/mL) | 3 | 377.6  (72.3) | 5 | 366.0  (30.5) | 3 | 492.3  (21.8) | 5 | 162.7  (45.3) | 0 | NC | 3 | 163.8  (60.8) | 8 | 262.2  (49.7) | 2 | 392.9  (NC) |
| C_ss, min_ (ng/mL) | 3 | 131.28  (28.9) | 5 | 171.55  (44.1) | 3 | 245.73  (28.3) | 5 | 54.33  (33.1) | 0 | NC | 3 | 65.16  (44.9) | 8 | 99.72  (61.0) | 2 | 174.65  (NC) |
| AUC_0-τ_ (ng•hr/mL) | 3 | 5628.2 (41.6) | 5 | 6744.8 (35.8) | 3 | 8518.0 (25.9) | 5 | 2614.7 (36.9) | 0 | NC | 3 | 2405.6 (49.5) | 8 | 4136.5 (51.8) | 2 | 6225.8 (NC) |
| **De-alkylated** |  |  |  |  |  |  |  |  |  |  |  |  |  |  |  |  |
| T_ss, max_ ^a^ (hr) | 3 | 7.92  (2.75-8.00) | 5 | 8.00  (6.00-8.45) | 3 | 4.02  (3.97-5.98) | 5 | 6.00  (4.05-12.00) | 0 | NC | 3 | 6.00  (5.97-6.32) | 8 | 4.03  (2.00-12.00) | 2 | 4.91  (3.88-5.93) |
| C_ss, max_ (ng/mL) | 3 | 23.91  (46.2) | 5 | 37.28  (11.7) | 3 | 35.08  (47.2) | 5 | 11.43  (57.1) | 0 | NC | 3 | 11.30  (89.1) | 8 | 18.20  (57.6) | 2 | 31.08  (NC) |
| C_ss, min_ (ng/mL) | 3 | 10.327  (48.1) | 5 | 20.973  (25.3) | 3 | 19.009 (44.3) | 5 | 4.131  (56.8) | 0 | NC | 3 | 3.243  (9.6) | 8 | 7.812  (60.8) | 2 | 18.313  (NC) |
| AUC_0-τ_ (ng•hr/mL) | 3 | 428.1  (37.5) | 5 | 746.0  (10.8) | 3 | 637.7  (41.1) | 5 | 196.2  (56.5) | 0 | NC | 3 | 140.6  (17.1) | 8 | 305.8  (55.2) | 2 | 531.4  (NC) |
| **Lactam** |  |  |  |  |  |  |  |  |  |  |  |  |  |  |  |  |
| T_ss, max_ ^a^ (hr) | 3 | 3.58  (2.75-8.00) | 5 | 8.00  (4.17-11.83) | 3 | 4.02  (3.97-5.98) | 5 | 6.00  (3.93-7.80) | 0 | NC | 3 | 6.00  (5.97-6.32) | 8 | 7.99  (3.97-12.03) | 2 | 4.91  (3.88-5.93) |
| C_ss, max_ (ng/mL) | 3 | 23.50  (33.3) | 5 | 17.94  (25.6) | 3 | 16.53  (57.7) | 5 | 11.94  (44.2) | 0 | NC | 3 | 12.29  (42.4) | 8 | 12.23  (25.0) | 2 | 15.11  (NC) |
| C_ss, min_ (ng/mL) | 3 | 7.924  (10.7) | 5 | 8.817  (31.9) | 3 | 7.770  (37.1) | 5 | 3.788  (36.2) | 0 | NC | 3 | 5.025 (38.3) | 8 | 4.897  (37.3) | 2 | 8.685  (NC) |
| AUC_0-τ_ (ng•hr/mL) | 3 | 364.1  (8.7) | 5 | 338.6  (26.8) | 3 | 273.4  (43.8) | 5 | 188.9  (30.9) | 0 | NC | 3 | 207.7  (48.5) | 8 | 214.7  (28.1) | 2 | 278.7  (NC) |

Abbreviations: T_ss_, _max_ = time to C_ss, max_; C_ss_, _max_ = observed maximum concentration at steady state in plasma; C_ss, min_= observed minimum concentration at steady state in plasma; AUC_0-τ_ = area under the concentration versus time curve during a dosing interval (24 hours) at steady state in plasma; CAD = concomitant anticancer drug; CV = coefficient of variation; NC = not calculated.

^a^ Median (minimum-maximum) is reported for T_ss, max_

Table S2

Summary of Geometric Mean (Geometric CV%) Pharmacokinetics Parameters for Vinorelbine with and without Epertinib (Arm B)

| **Dose of epertinib** | ***N*** | **200 mg**  ***N* = 5** | ***N*** | **400 mg**  ***N* = 2** |
| --- | --- | --- | --- | --- |
| **Epertinib absent** |  |  |  |  |
| C_max_ (ng/mL) | 5 | 77.1 (77.8) | 2 | 40.4 (NC) |
| T_max_^a^ (hr) | 5 | 2.00 (1.00-2.00) | 2 | 1.70 (1.00-2.40) |
| AUC_0-t_ (ng•hr/mL) | 5 | 358.0 (60.2) | 2 | 222.5 (NC) |
| AUC_0-inf_ (ng•hr/mL) | 5 | 400.4 (61.2) | 2 | 262.1 (NC) |
| **Epertinib present** |  |  |  |  |
| C_max_ (ng/mL) | 4 | 47.6 (84.4) | 0 | NC |
| T_max_^a^ (hr) | 4 | 1.49 (0.83-3.93) | 0 | NC |
| AUC_0-t_ (ng•hr/mL) | 4 | 267.1 (50.5) | 0 | NC |
| AUC_0-inf_ (ng•hr/mL) | 4 | 296.8 (52.6) | 0 | NC |

Abbreviations: C_max_ = observed maximum concentration in plasma; T_max_ = time to C_max_; AUC_0‑t_ = area under the concentration versus time curve from time zero (pre-dose) to the last quantifiable concentration in plasma ; AUC_0-inf_ = area under the concentration versus time curve from zero (pre-dose) extrapolated to infinity; CV = coefficient of variation; NC = not calculated.

^a^ Median (minimum-maximum) is reported for T_max_.

Table S3

Summary of Geometric Mean (Geometric CV%) Pharmacokinetics Parameters for Capecitabine with and without Epertinib (Arm C)

| **Dose of epertinib** | ***N*** | **200 mg**  ***N* = 4** | ***N*** | **400 mg**  ***N* = 9** | ***N*** | **600 mg**  ***N* = 4** |
| --- | --- | --- | --- | --- | --- | --- |
| **Epertinib absent** |  |  |  |  |  |  |
| C_max_ (µg/mL) | 3 | 0.1087 (209.4) | 7 | 0.0847 (66.4) | 3 | 0.1272 (65.9) |
| T_max_^a^ (hr) | 3 | 1.10 (1.00-2.00) | 7 | 1.03 (0.55-11.87) | 3 | 2.58(1.98-8.00) |
| AUC_0-t_ (µg•hr/mL) | 3 | 0.2182 (101.9) | 7 | 0.2242 (74.3) | 2 | 0.5250 (NC) |
| AUC_0-inf_ (µg•hr/mL) | 0 | NC | 2 | 0.302 (NC) | 0 | NC |
| **Epertinib present** |  |  |  |  |  |  |
| C_max_ (µg/mL) | 3 | 0.1375 (144.3) | 7 | 0.0927 (50.9) | 2 | 0.1531 (NC) |
| T_max_^a^ (hr) | 3 | 2.00 (2.00-4.03) | 7 | 2.00 (1.00-3.98) | 2 | 1.21 (0.45-1.97) |
| AUC_0-t_ (µg•hr/mL) | 3 | 0.3906 (110.9) | 7 | 0.2260 (23.7) | 1 | 0.2330 (NC) |
| AUC_0-inf_ (µg•hr/mL) | 0 | NC | 2 | 0.288 (NC) | 1 | 0.236 (NC) |

Abbreviations: C_max_ = observed maximum concentration in plasma; T_max_ = time to C_max_; AUC_0‑t_ = area under the concentration versus time curve from time zero (pre-dose) to the last quantifiable concentration in plasma; AUC_0-inf_ = area under the concentration versus time curve from zero (pre-dose) extrapolated to infinity; CV = coefficient of variation; NC = not calculated.

^a^ Median (minimum-maximum) is reported for T_max_.

Table S4

Summary of Geometric Mean (Geometric CV%) Pharmacokinetics Parameters for 5-Fluorouracil following treatment with Capecitabine with and without Epertinib (Arm C)

| **Dose of epertinib** | ***N*** | **200 mg**  ***N* = 4** | ***N*** | **400 mg**  ***N* = 9** | ***N*** | **600 mg**  ***N* = 4** |
| --- | --- | --- | --- | --- | --- | --- |
| **Epertinib absent** |  |  |  |  |  |  |
| C_max_ (µg/mL) | 3 | 0.1087 (209.4) | 7 | 0.0847 (66.4) | 3 | 0.1272 (65.9) |
| T_max_^a^ (hr) | 3 | 1.10 (1.00-2.00) | 7 | 1.03 (0.55-11.87) | 3 | 2.58 (1.98-8.00) |
| AUC_0-t_ (µg•hr/mL) | 3 | 0.2182 (101.9) | 7 | 0.2242 (74.3) | 2 | 0.5250 (NC) |
| AUC_0-inf_ (µg•hr/mL) | 0 | NC | 2 | 0.302 (NC) | 0 | NC |
| **Epertinib present** |  |  |  |  |  |  |
| C_max_ (µg/mL) | 3 | 0.1375 (144.3) | 7 | 0.0927 (50.9) | 2 | 0.1531 (NC) |
| T_max_^a^ (hr) | 3 | 2.00 (2.00-4.03) | 7 | 2.00 (1.00-3.98) | 2 | 1.21 (0.45-1.97) |
| AUC_0-t_ (µg•hr/mL) | 3 | 0.3906 (110.9) | 7 | 0.2260 (23.7) | 1 | 0.2330 (NC) |
| AUC_0-inf_ (µg•hr/mL) | 0 | NC | 2 | 0.288 (NC) | 1 | 0.236 (NC) |

Abbreviations: C_max_ = observed maximum concentration in plasma; T_max_ = time to C_max_; AUC_0‑t_ = area under the concentration versus time curve from time zero (pre-dose) to the last quantifiable concentration in plasma; AUC_0-inf_ = area under the concentration versus time curve from zero (pre-dose) extrapolated to infinity; CV = coefficient of variation; NC = not calculated.

^a^ Median (minimum-maximum) is reported for T_max_.
